# Evaluation of the nutritional status of rural children living in Zambia

**DOI:** 10.1186/s40101-020-00244-8

**Published:** 2020-11-16

**Authors:** Arimi Mitsunaga, Taro Yamauchi

**Affiliations:** 1 Graduate School of Health Sciences, Hokkaido University, Sapporo, Japan; 2 Faculty of Health Sciences, Hokkaido University, N12-W5, Kitaku, Sapporo, 060-0812 Japan

**Keywords:** Zambia, Secular trend, Growth chart, Nutritional status

## Abstract

**Background:**

Growth references (growth charts) are used to assess the nutritional and growth status of children. In developed countries, there are growth charts based on the country’s national surveys. However, many developing countries do not have their own growth references, so they usually use WHO and US CDC references. In such cases, it is known that the growth statuses of the subject are underestimated or overestimated. This study sought to accurately assess the growth status of children by developing a local growth chart for children living in the Southern Province of Zambia.

**Methods:**

Anthropometric measurements were conducted on 1135 children aged 2–19 years in the target area. The height and weight data for each sex and age were smoothed using the LMS method, and a percentile curve of height and weight was developed. Based on the US CDC reference, three indicators of undernutrition (stunting, underweight, and thinness) were calculated to determine the proportion of those who are undernourished (*z*-score < − 2).

**Results:**

The 50th percentile curve of height and weight in the growth chart of the target population was equivalent to the 5–25th percentile curve of the US reference, and the children of Zambia were both small and of low weight. Conversely, although many subjects are small and of low weight, it was found that there were few poor nutritional statuses as judged by BMI. Through comparison with a previous study in the Southern Province of Zambia, a secular change in the growth of children over 20 years was found. Although there was no significant increase in height and weight in the older age group, there was a significant increase in height and weight at an earlier age. This is thought to be due to an increase in the growth rate of children due to improved socioeconomic conditions, women’s education level, and improvement in health care standards.

**Conclusions:**

The subjects were small and of low weight compared to the US population, but the nutritional status was not poor by BMI. The height and weight at an earlier age increased compared to 20 years ago.

**Supplementary Information:**

The online version contains supplementary material available at 10.1186/s40101-020-00244-8.

## Background

Child growth is internationally recognized as an important indicator for monitoring nutritional status and health in populations [1]. Poor circumstances in early childhood are associated with an increased risk of chronic diseases and their underlying risk factors, which affect life-long health [2, 3].

Undernutrition in developing countries has been an internationally important issue, as exemplified by the launch of the Scaling up Nutrition (SUN). In particular, previous research [4] described that sub-Saharan Africa has an undernutrition problem. In Zambia, located in sub-Saharan Africa, the rates of stunting and underweight for children under 5 years were 45% and 15%, respectively, according to the national investigation [5]. This means that in Zambia, child undernutrition is a problem, as in other sub-Saharan countries.

One of the important events in considering the nutritional status of Zambian children is the relocation of people for the construction of the Kariba Dam during the late 1950s to the early 1960s. Children who were forced to relocate for the dam construction were surveyed on nutritional status and growth before relocation (1957–1958) and about 35 years later (1993) [6, 7]. Comparing children aged from 8 to 13 years between two surveys, it was concluded that there was no difference in the height and weight of children in rural areas and that there was no secular change in about 35 years from the late 1950s to the early 1990s [6, 7]. However, since the early 1990s, there has been no research on the growth of children in the region, and the secular change in the growth of children over the past 20 years from the early 1990s to the present is unclear.

Generally, growth references are used by doctors and nurses involved in the care of individual children in medical assessment to evaluate the growth status of a child. Furthermore, growth references are used as a public health tool to summarize and compare anthropometric data among groups of children [8]. While an international reference may be needed for comparison of height and weight between regions or countries, local references best reflect the characteristics of the population. Therefore, the current trend in many countries is to develop their local references or for use in the clinic [9]. Nevertheless, there are no national-level growth references in Zambia, and there are limited studies on the growth chart of children, such as Gillett (1995) and Gillett and Tobias (2002) [6, 7].

Therefore, this study aimed at the following three points to evaluate the growth status of children living in the Southern Province of Zambia: (1) develop a local growth chart and compare it with international references and previous studies in other Saharan countries, (2) evaluate the growth status by focusing on sex differences, and (3) compare this with previous research [6] in the same region 20 years ago to ascertain whether there was a change in the growth of children living in the Southern Province of Zambia.

## Methods

### Study area

The Republic of Zambia is located in a semi-arid tropical zone in the southern part of Africa. It has two seasons: a rainy season (December to March) and a dry season (April to November). The average elevation within the country is approximately 1300 m, and the annual rainfall is about 500–1500 mm. Traditional farming in rural villages depends on rainfall, and food production is likely to be affected by climatic variations such as drought or heavy rain.

Zambia is the second-largest copper producer in Africa and achieved middle-income country status in 2011. The human development index in 2011 was 0.430, life expectancy at birth was 49.0, and population living below the national poverty line was 59.3% [10]. For socioeconomic status, 94.0% of primary school-age children enrolled in primary education [11]; 48% of women and 49% of men engaged in agricultural occupations [12].

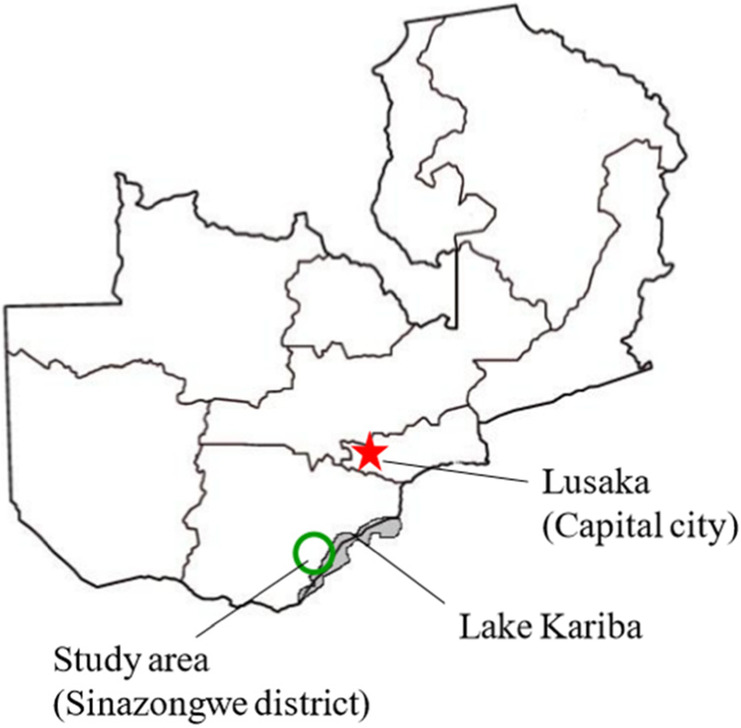



The study area is located in the Southern Province of Zambia

### Subjects

The survey included 1135 children (576 boys, 559 girls) who lived in the Sinazongwe district in the Southern Province of Zambia. Anthropometric measurements were taken of children attending five primary schools near the city of Sinasese. These five primary schools and children were randomly selected. Among the subjects, the preschoolers (2 to 6 years old) and older children who had quit or graduated from school lived in four villages around the school (7 to 19 years old). All children assigned ages from 1.5 to 2.4 were grouped as 2 years old. Another age group was also determined according to this approach. Pregnant and lactating girls were excluded from the study because of the weight effects of these conditions. Our sample has the same ethnicity which is called Tonga people and the same socioeconomic characteristics like property, family structure, and business. For instance, their houses were brick and with a straw-thatched roof. Furthermore, our sample’s mothers were mainly housewives, and they had several siblings.

The main livelihood of the study area is agriculture, which is supplemented by animal husbandry, hunting and gathering, and cash earning. The market that is the easiest to access from the study area is about an hour’s walk away. Daily necessities can be purchased in the town. Maize is mainly cultivated as a staple food crop in the study area, and it is cooked in various forms and eaten. Especially for “Nshima,” made from ground maize powder and hot water, it is observed the most.

### Measurements

All anthropometric measurements, including height and weight, were conducted by an experienced researcher using standard procedures [13]. Height was measured to the nearest 0.1 cm using a portable stadiometer (model 213 Seca, Germany). Weight was measured to the nearest 0.1 kg using a digital scale (TANITA, Japan). From the data, body mass index (BMI, kg/m^2^) was calculated. The data were collected twice, from August to September 2011 and August 2012. At this time, subjects measured in 2011 were excluded from the study in 2012.

The age of the subjects was determined based on the “under 5-year clinic card” and/or the children’s knowledge, which was confirmed or revised based on the information provided by the school’s attendance book. Through this information, we could confirm the date of birth of the subjects and clarify the exact age of them. Children who did not know their date of birth were also confirmed with their mothers.

The study was approved by the Ethics Committee of the Graduate School of Health Sciences, Hokkaido University. Participation in the study was voluntary, after the study had been explained to the participants and care persons in local languages. Consent was obtained from the children, their parents, and/or the schoolmaster and teachers.

### Curve smoothing

**Fig. 1:**
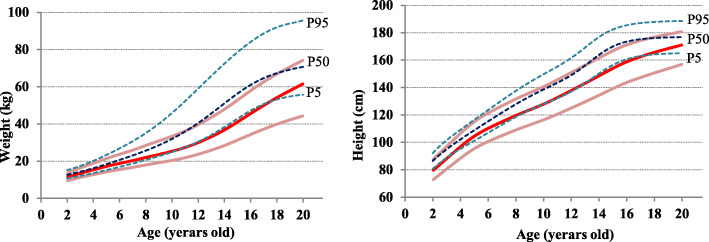
Comparison of height and weight percentile curves for rural Zambian boys with the CDC reference (*n* = 576)

**Fig. 2:**
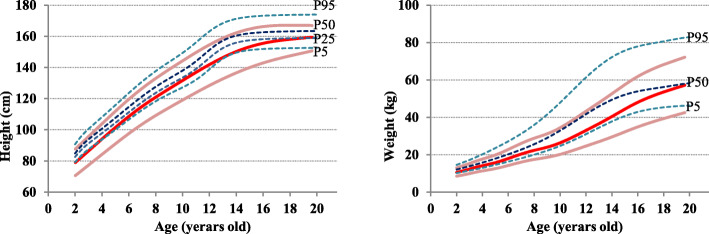
Comparison of height and weight percentile curves for rural Zambian girls with the CDC reference (*n* = 559)

The LMS method [14] was used to develop sex-specific smoothed growth curves for height and weight. The LMS method is a mathematical smoothing method that involves a process to normalize the original data using a Box-Cox transformation. This method contains three distribution curves representing the median (*M*), coefficient variance (*S*), and skewness (*L*) as a Box-Cox power. The fitting process was performed by changing these three values (*L*, *M*, and *S*) to smooth the data across each age and standardize the original dataset as a normal distribution. The goodness of fit of the curve was established using the *Q* test [15, 16] and worm plotting [17]. The LMS Chart Maker Pro ver.2.2 program (Medical Research Council, UK) developed the LMS model. Three smoothing curves of 5th, 50th, and 95th percentiles were developed for the height and weight of Zambian children and compared to the corresponding percentile curves of the US reference [18]. For girls’ height, the smoothing curves of the 25th percentile were added (Figs. 1 and 2).

The height-for-age *z*-score (HAZ), weight-for-age *z*-score (WAZ), and BMI-for-age *z*-score (BMIAZ) were calculated using the Center for Disease Control and Prevention (CDC) 2000 reference [18]. A *z*-score below − 2 was defined as undernutrition [19], stunting (HAZ < − 2), underweight (WAZ < − 2), and thinness (BMIAZ < − 2). Additionally, the growth status of the subjects was evaluated by determining the *z*-score.

For age-specific height and weight, the subject’s data were compared to the literature data [6, 7] using the *Z* test. The existence of secular change in children’s height and weight was confirmed.

All values are shown as mean ± standard deviation (SD). All statistical analyses were performed using the JMP 10.0.2 software package (SAS Institute Inc.). The level of statistical significance was set at *P* < 0.05.

## Results

### Growth patterns

In boys, the height and weight curves for Zambian children almost corresponded with the 5th percentile of the CDC curves. In girls, the height curves were positioned between the 5th and 25th percentiles of the CDC curves and weight curves positioned between the 5th and 50th percentiles of the CDC curves. The average height at the age of 19 was 168.9 ± 7.3 cm for boys and 156.4 ± 3.5 cm for girls (Table 2). Compared to the results of previous studies on adults in the same group (males 165.3 ± 9.0 cm, females 156.7 ± 5.5 cm [20]), boys were slightly taller, and girls were comparable. Conversely, the average weight at the age of 19 was 59.4 ± 5.6 kg for boys and 53.8 ± 2.9 kg for girls, which was heavier for both men and women compared to previous studies (males 54.3 ± 9.1 kg, females 51.5 ± 7.5 kg [20]).

**Fig. 3:**
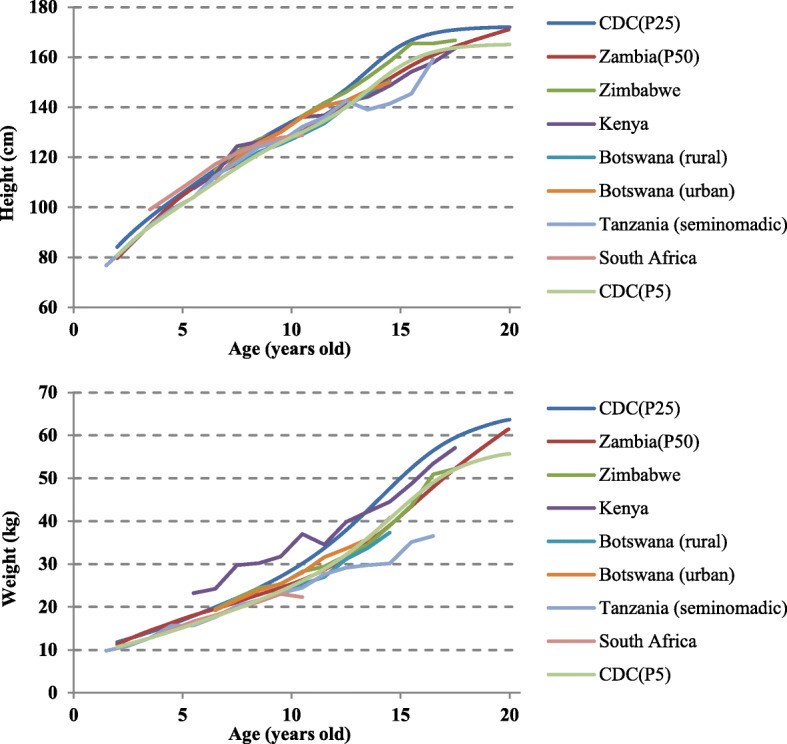
The mean height and weight of boys compared with subjects from different sub-Saharan countries

**Fig. 4:**
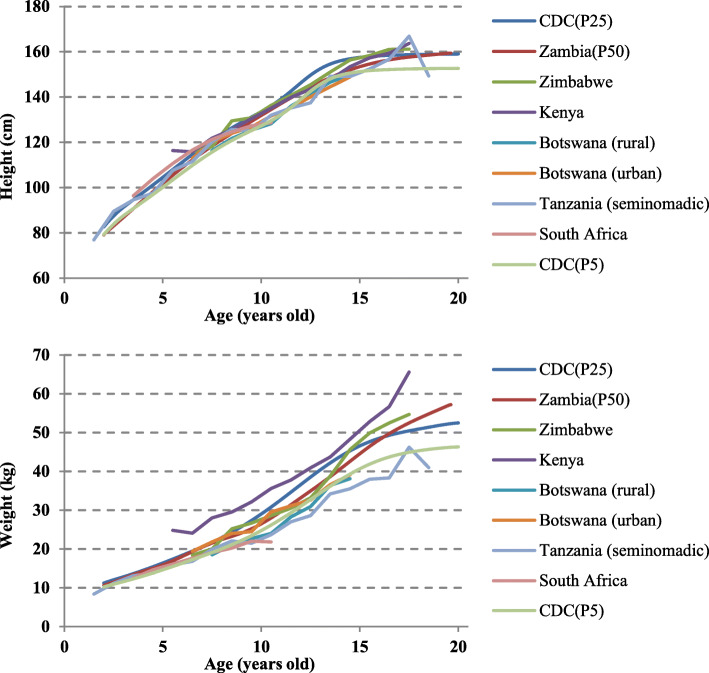
The mean height and weight of girls compared with subjects from different sub-Saharan countries

The height and weight growth curves (50th percentile) of the target population are compared with the US reference [18] and six sub-Saharan countries [21–26] (Botswana [urban], Botswana [rural], Kenya, South Africa, Tanzania, Zimbabwe) in Figs. 3 and 4. Except for Kenya, both men and women were below the 25th percentile of the US reference in height and weight. The growth curves of the height and weight of the populations in the sub-Saharan countries were similar (Figs. 3 and 4).

### Growth status

Table 1 shows the average of the three *z*-scores, standard deviation, and percentage of those with undernutrition (*z*-score < − 2). “Stunting” (HAZ < − 2) was the highest percentage in both sexes, followed by “underweight” (WAZ < − 2). Conversely, the ratio of a low BMI (thinness: BMIAZ < − 2) was less than 10%, which was lower than the other two indicators. In other words, although many subjects were small (stunting) and of low weight (underweight), it was found that many subjects were normal as judged by BMI. Among all indicators, boys had a higher percentage of undernutrition than girls, and sex differences were seen in nutritional status, but there were no significant differences.

**Table 1:** Sex- and age-specific *z*-score (HAZ, WAZ, BMIAZ) for rural Zambian children

	*n*	Boys			*n*	Girls			*P*
		Mean	SD	*z* < − 2 (%)		Mean	SD	*z* < − 2 (%)	
HAZ	576	− 1.57	1.20	36.5	559	− 1.31	1.28	26.5	< 0.001
WAZ	576	− 1.42	1.24	30.4	559	− 1.12	1.28	19.7	< 0.0001
BMIAZ	576	− 0.53	1.12	7.1	559	− 0.30	1.02	5.2	< 0.001

*HAZ* height-for-age *z*-score, *WAZ* weight-for-age *z*-score, *BMIAZ* BMI-for-age *z*-score, *SD* standard deviation

### Secular change

Sex- and age-specific growth data (mean and SD) of this study were compared to those of a previous study (measured in 1993 [6] and measured in 1957–1958 [7] in Tables 2 and 3). The subjects of this study were taller and heavier than children in the region 2 years ago except for 9 years, 10 years, and 16 to 19 years in boys and 9 and 17 to 19 years in girls. Boys’ height was significantly higher than that of previous subjects, except for boys at 9 years, 10 years, and 16 to 19 years. Similarly, for girls, except for the ages of 9 and 17 to 19 years, it was significantly higher than in previous subjects. Conversely, in terms of body weight, boys were significantly heavier than previous subjects, except for those at 10 years of age, and girls were significantly heavier, except 18 and 19-year-olds.

**Table 2:** Mean height and weight of boys compared with subjects from previous Zambian study

Age	*n*	2011–2012, mean (SD)	*n*	1993^a^, mean (SD)	*n*	1957–1958^b^, mean (SD)
Boy’s height						
2	40	84.6 (5.4)	35	81.1 (3.6)*		
3	31	91.5 (6.5)	33	87.2 (4.5)*		
4	38	101.6 (7.1)	26	94.5 (3.4)*		
5	32	108.2 (6.3)	40	100.6 (4.7)*		
6	24	112.8 (7.1)	35	107.7 (4.4)*		
7	25	116.6 (7.4)	54	113.2 (7.1)*	3	129.8 (5)*
8	24	122.6 (5.8)	61	116.9 (5.6)*	2	124.2 (2.9)*
9	23	125.1 (6.1)	109	124.7 (6.9)	25	124.9 (6.6)
10	24	129.3 (5.7)	61	129 (6.9)	15	127.7 (4.6)
11	50	135.6 (8.6)	49	131.8 (5.3)*	7	132.1 (7.1)*
12	30	140.6 (8.6)	71	136.9 (7.2)*	34	135.7 (8.5)*
13	32	145.5 (10.4)	48	137.7 (6.6)*	2	142.3 (9.2)*
14	50	150.0 (9.4)	48	144.8 (8.0)*		
15	47	156.0 (9.1)	26	152.7 (9.1)*		
16	33	160.0 (8.4)	44	157.5 (10.0)		
17	30	163.4 (9.0)	69	161.8 (8.1)		
18	29	167.5 (6.0)	18	165.8 (6.7)		
19	14	168.9 (7.3)	30	166.4 (5.6)		
Boy’s weight						
2	40	12.5 (1.5)	35	10.4 (1.2)*		
3	31	14.7 (2.0)	33	12.5 (1.6)*		
4	38	16.1 (2.3)	26	13.9 (1.3)*		
5	32	18.5 (2.0)	40	14.8 (1.5)*		
6	24	19.7 (3.2)	35	17 (2.2)*		
7	25	21.0 (2.8)	54	19.2 (2.8)*	3	26.5 (1.4)*
8	24	23.7 (3.6)	61	20.6 (2.9)*	2	23.4 (3.5)
9	23	24.7 (3.5)	109	23.2 (3.1)*	25	26 (4.9)
10	24	25.8 (2.6)	61	25.5 (3.9)	15	25.2 (2.2)
11	50	29.5 (6.1)	49	26.5 (2.9)*	7	27.8 (3.8)*
12	30	33.1 (4.8)	71	30.3 (5.0)*	34	31.6 (6.9)
13	32	34.8 (7.1)	48	30.4 (4.4)*	2	33.5 (5.9)
14	50	38.6 (7.3)	48	33.7 (5.5)*		
15	47	43.9 (7.6)	26	39.6 (6.8)*		
16	33	48.0 (7.6)	44	44.2 (9.7)*		
17	30	52.6 (9.6)	69	49.7 (7.9)*		
18	29	57.3 (5.8)	18	53.5 (7.5)*		
19	14	59.4 (5.6)	30	54.6 (8.2)*		

^a^Gillet [6]

^b^Gillet and Tobias [7]

*P < 0.05, by *Z* test

**Table 3:** Mean height and weight of girls compared with subjects from previous Zambian study

Age	*n*	2011–2012, mean (SD)	*n*	1993^a^ ,mean (SD)	*n*	1957–1958^b^, mean (SD)
Girl’s height						
2	54	82.2 (5.8)	32	79.1 (2.8)*		
3	26	91.1 (5.4)	33	86.1 (4.4)*		
4	36	97.7 (6.1)	32	92.5 (4.7)*		
5	25	104.8 (8.5)	29	101 (4.5)*		
6	25	114.9 (7.1)	37	106 (6.9)*		
7	25	120.3 (7.6)	71	112.3 (5.8)*		
8	34	123.2 (5.3)	71	117.5 (5.9)*	7	119.3 (5.4)*
9	31	127.0 (5.7)	110	123.9 (6.9)	18	119.7 (3.7)*
10	30	133.4 (9.1)	89	128.8 (6.0)*	11	126.1 (7.4)*
11	35	139.0 (8.6)	81	133.2 (6.9)*	14	127.2 (5.0)*
12	37	144.1 (8.0)	92	135.3 (6.9)*	8	130.5 (9.9)*
13	37	148.5 (10.4)	38	142.8 (7.1)*	19	133.9 (6.8)*
14	38	151.4 (9.5)	43	148.2 (6.8)*		
15	37	155.7 (7.4)	36	151.6 (5.7)*		
16	35	155.8 (5.2)	33	152.7 (6.2)*		
17	28	158.1 (6.6)	36	156.8 (5.2)		
18	22	156.5 (5.2)	12	157.4 (7.1)		
19	4	156.4 (3.5)	16	159.4 (5.1)		
Girl’s weight						
2	54	11.4 (1.7)	32	9.4 (1.1)*		
3	26	14.0 (1.8)	33	12 (1.4)*		
4	36	14.9 (1.6)	32	13.5 (1.4)*		
5	25	17.0 (2.7)	29	15.1 (0.9)*		
6	25	19.5 (3.0)	37	16.6 (2.9)*		
7	25	22.6 (4.2)	71	17.9 (2.9)*		
8	34	23.4 (3.2)	71	20.5 (2.6)*	7	21.3 (2.8)*
9	31	24.5 (3.1)	110	22.7 (3.4)*	18	22.1 (2.3)*
10	30	29.0 (5.8)	89	25.3 (3.2)*	11	24.9 (3.6)*
11	35	31.4 (5.1)	81	27.3 (4.2)*	14	25.7 (4.1)*
12	37	35.3 (7.1)	92	28.6 (4.4)*	8	26.6 (4.8)*
13	37	38.9 (8.4)	38	33.3 (5.5)*	19	29.2 (4.7)*
14	38	41.4 (8.9)	43	37.7 (6.3)*		
15	37	47.7 (7.8)	36	40.3 (5.3)*		
16	35	49.8 (7.9)	33	43.8 (6.6)*		
17	28	54.0 (9.5)	36	50.5 (6.6)*		
18	22	52.7 (6.2)	12	51.8 (7.0)		
19	4	53.8 (2.9)	16	54.5 (5.5)		

^a^Gillet [6]

^b^Gillet and Tobias [7]

*P < 0.05, by *Z* test

## Discussion

Local references best reflect the current ethnic characteristics of a particular population [9]. By developing and adapting local references, the growth status of children living in the population was assessed. The production of local reference standards in a developing country has many difficulties, such as the lack of materials and skills of the measurer to conduct a highly accurate anthropometry survey; therefore, the absence of reliability would be a severe problem. In this survey, an experienced researcher conducted anthropometric measurements using a highly accurate stadiometer and scale. Furthermore, by using the LMS method, which can draw smoothed growth curves [14], a reference chart was developed.

### Growth status from the growth chart and *z*-score

The 50th percentile curve of the target population’s height and weight corresponds to the 5–25th percentile of the US CDC reference (Figs. 1 and 2), and the children of the Southern Province of Zambia were generally recognized to have short height and low weight. This was also the case in previous studies in sub-Saharan countries [21–26] (Figs. 3 and 4).

Indeed, one fifth to one third of the subjects were determined to be “stunted” (HAZ < − 2) and “underweight” (WAZ < − 2), while those with a low BMI were below 10%. BMI is an indicator of overall nutritional status. In other words, although the subjects were small and of low weight compared to the US CDC references, they had sufficient weight for their height, suggesting that their growth status was not poor. Of course, we understood that there are limitations of BMI as a nutritional status assessment tool; for instance, BMI does have limitations in its ability to assess adiposity, and its use in actual clinical practice is quite limited [27]. However, BMI has strengths in that it is cheap and relatively easy to use. Therefore, in this survey, it was appropriate to use BMI to assess the growth status of children because we were in a situation of limited resources.

In Figs. 3 and 4, we compared Zambian children to 6 areas in sub-Saharan countries, and it revealed that the growth curves of height and weight of the populations in the sub-Saharan countries were similar and Zambian children are almost in middle status. Therefore, it was found that the growth status of Zambian children was roughly moderate in all sub-Saharan countries (Figs. 3 and 4). The height and weight curves of sub-Saharan countries were below the 25th percentile of the CDC curves. Generally, sub-Saharan children were recognized as “smaller” than US children because of the retardation of growth velocity [21] and the effects of poor living conditions [26].

### Sex differences in growth status

Regarding the sex differences, in the three indicators of undernutrition (stunting, underweight, and thinness), the proportion of undernourished children was higher in boys than in girls. A similar trend has been reported in previous studies in other sub-Saharan countries such as Kenya [23] and Nigeria [28].

The reasons why boys showed a higher undernutrition rate than girls were (1) differences in occupation activity in traditional society and (2) biological differences in sexes. In traditional society, adults tend to label boys as more sturdy, self-confident, and self-reliant, whereas girls are labeled as more fragile, fearful, and dependent [29].

This recognition might have affected the sex differences in occupation activity in traditional society and caused definitive differences in nutritional status between boys and girls. In traditional society, boys are more involved in heavy work activities such as gathering vegetable foods, trapping rabbits and hares, and killing birds [24], whereas girls had greater access to food because they helped with cooking [23]. Consequently, it is suggested that boys are relatively large in energy consumption, and simultaneously, women’s food intake increases, which results in sex differences in indicators of undernutrition.

Biologically, in sub-Saharan Africa, male children are consistently more likely to become “stunted” than females, which might suggest that males were more vulnerable than females [30]. Male physiology in early life is inherently less robust than that of females [31], which implies the biological vulnerability of boys. According to the Zambia Demographic and Health Survey (ZDHS), the mortality rate for children under 5 years of age in 1992 was 188 males and 168 females per 1000 live births, indicating a higher male mortality rate [32]. In 2007, the mortality rate decreased to 151 boys and 124 girls, but still showed higher mortality rates than boys [5].

Due to the social and biological differences such as livelihood activities, behavioral patterns, and biological vulnerabilities, boys were probably relatively undernourished than girls.

### Secular change

There was no doubt that secular change in height, weight, and growth tempo occurred over long periods [33]. Previous research [34] describing the Bantu in Cameroon increase in adult height during the last 50 years most likely reflects improvements in hygienic conditions and better nutrition. The history of the growth study of children started from the late 1950s to the early 1960s. In Zambia, during the late 1950s to the early 1960s, people were forced to relocate because of the construction of the Kariba Dam. Children who were forced to relocate due to dam construction were surveyed on nutritional status and growth before relocation (1957–1958) and about 35 years later (1993) [6, 7]. Comparing children aged from 8 to 13 years between two surveys, it was concluded that there was no difference in the height and weight of children in rural areas and that there was no secular change in roughly 35 years from the late 1950s to the early 1990s [7]. This study uses child height and weight data from 1993 [6] as a baseline and examined secular change. Furthermore, height and weight data from 1957 to 1959 [7] were also included.

Height reflects long-term nutritional status, while weight is affected by short-term nutritional status [19]. Therefore, it is more suitable for height than weight to consider the secular change for growth. Compared to 20 years ago, adult height did not change in either sex, but the average height of boys aged 11–15 years (boys) and 10–16 years (girls) increased significantly. This result suggests that the growth pattern of children has changed over the last 20 years; that is, the growth rate is faster than 20 years ago. Furthermore, we found an increase in BMI of all age groups except for the age of 4 and 6 years in girls. Therefore, it can be observed that there are improvements in nutrition in 20 years from the early 1990s to 2011–2012.

The present study suggested that the age to reach the adult value (males 165.3 cm, female 156.7 cm [20]) was 17 to 18 years (boys) and 16 to 17 years (girls). In the early 1990s, in the Southern Province of Zambia, the age of menarche was 15.3 years, and the age of testicular maturation was 17.1 years [35, 36]. In general, it is known that there were 4- to 5-year gaps between the age of menarche or testicular maturation and reaching adult height [37]. If the growth tempo had not changed over the 20 years, it is possible to estimate that the age of reaching adult height would be almost 20 years old in both sexes (boys 17.1 + 4 = 21.4, girls 15.3 + 4 = 19.3). Nevertheless, in the present study, the age of reaching adult height was 17 to 18 years (boys) and 16 to 17 years (girls), as mentioned. In other words, it was suggested that the growth tempo became faster in both sexes compared to 20 years ago.

It is known that socioeconomic positions and the mother’s education are associated with the stature of children [38]. According to the ZDHS in 1997 and 2002, the percentage of women who did not receive education decreased from 27.0 to 13.2%. In other words, women’s education level has improved in 15 years. The socioeconomic status in southern provinces changed drastically over the last 20 years, judging from an example of the maintenance of infrastructure and the rapid spread of mobile phones in villages. Furthermore, due to the spread of vaccination for infants and the improvement of medical facilities, under-five mortality rate decreased from 197 to 75 within 20 years (1996–2014) [12]. Also, it is supposed that the hygiene environment advanced in this region compared to 20 years ago because the proportion of the population without access to improved sanitation decreased drastically from 38 to 13% within 20 years [39]. Based on these factors, the prevalence of infectious diseases is presumed to decrease. The percentage of “stunting” for under 5 years old was 46% in 1992 and 45% in 2007, which was almost the same for 15 years. However, the percentage of “underweight” decreased from 21 to 15% [5]. The surveyed population increased in growth rate compared to the previous 1993 results of the region but did not increase adult height. However, in the case of Cameroon described above, adult height and weight were compared for 50 years, whereas in this study, only a 20-year comparison was conducted. A previous study [40] described that secular change in adult men and women takes longer than that of children. Therefore, if the socioeconomic situation and the health/sanitary environment improved steadily, it is expected that adult height will increase.

Secular increase in height seems to be slowing down at the age of 9 to 10 in boys, although we can confirm secular trends generally. There are several studies which confirmed the slowing down of secular increase in height [41], and environmental constraints were related to them [42, 43]. We consider that the reason is related to severe drought and famine in Southern Africa in 2002. The food security in the Southern Africa region was at its lowest level since 1992. It can be expected that the children who were born during this severe drought and famine and pregnant women could not receive enough amount of nutritious food. It means that secular increase in height seems to be slowing down at age of 9 to 10 in boys can be associated to this severe drought and famine.

Generally, boys are more susceptible to the environment than girls. Therefore, it is speculated that boys are easily affected by severe malnutrition immediately after birth and in the prenatal period than girls. In this reason, we can expect that only boys showed slowing down of the secular trend in 9 and 10 years old.

We can use this logic to explain the secular increase in height in boys of around 2.5 cm in adolescent and adult height, which could not be observed in girls. In other words, this is typical in developing countries that growth of boys is more influenced by the environmental situation than the growth of girls [44].

### Limitation of survey

A major limitation of the study was the small number of girls aged 19 years. As girls of this generation often entered the pregnancy and lactation period, there were many cases in which an anthropometric survey could not be conducted. Nevertheless, by 19 years of age, children in both sexes in the present study had reached the adult height and weight of adult Zambian; it is conceived that the references that developed during the present study have some reliability.

Another limitation of the study was the lack of information of peak height velocity (PHV). If we have this information, we could observe a sign of accelerated developmental tempo and we could confirm the sign of puberty. However, in the previous study which we have compared, PHV was not discussed. Therefore, we can say that we have conducted sufficient research from the perspective of comparing secular change.

Yamauchi and Kon [45] described that a person living in a target area might change their body weight over 1 year with the agricultural cycle during the agricultural season, harvest season, and idle season. In this survey, cross-sectional data were used in the dry season (August to September), and it is difficult to consider the weight change due to seasonality. However, seasonality has little effect on height, and the growth reference developed in the present study is useful for assessing the nutritional status and growth of children in the target area. It is hoped that a comprehensive understanding of the nutritional status of children living in the target area will be pursued by investigating foods and physical activities that have a major impact on the nutritional status and growth of children.

## Conclusions

A growth chart was developed from sex- and age-specific anthropometry data, and the subjects were 1115 children (2 to 19 years old). The subjects were small and of low weight compared to the US population, but the growth status was not poor as judged by BMI.

Similar to a previous study on sub-Saharan Africa, boys had a higher undernutrition rate than girls in all HAZ, WAZ, and BMIAZ. The reasons for this are the differences in occupation activity in traditional society and biological differences between sexes.

Compared to 20 years ago, secular change was seen in the physique of children, and the height and weight at an earlier age increased.

## Supplementary Information

**Additional file 1.**

## Data Availability

The datasets used and/or analyzed during the current study are available from the corresponding author on reasonable request.

## Abbreviations

SUN: Scaling Up Nutrition
BMI: Body mass index
HAZ: Height-for-age *z*-score
WAZ: Weight-for-age *z*-score
BMIAZ: BMI-for-age *z*-score
CDC: Center for Disease Control and Prevention
SD: Standard deviation
ZDHS: Zambia Demographic and Health Survey

## References

[1] de Onis M, Blossner M (2003). The World Health Organization Global Database on Child Growth and Malnutrition: methodology and applications. Int J Epidemiol.

[2] Grantham-Mcgregor S (1995). A review of studies of the effect of severe malnutrition on mental development. J Nutr.

[3] Thomas D, Frankenberg E (2002). Health, nutrition and prosperity: a microeconomic perspective. Bull World Health Organ..

[4] Nube M, Sonneveld B (2005). The geographical distribution of underweight children in Africa. Bull World Health Organ..

[5] Central Statistical Office (2009). Zambia Demographic and Health Survey 2007.

[6] Gillett RM (1995). Growth and physical status: biocultural measures of long-term underdevelopment among the Gwembe Tonga of Zambia. Doctoral Dissertation.

[7] Gillett RM, Tobias PV (2002). Human growth in southern Zambia: a first study of Tonga children predating the Kariba Dam (1957-1958). Am J Hum Biol..

[8] Kulaga Z, Litwin M, Tkaczyk M, Rozdzynska A, Barwicka K, Grajda A (2010). The height-, weight-, and BMI-for-age of Polish school-aged children and adolescents relative to international and local growth references. BMC Public Health.

[9] Neyzi O, Furman A, Bundak R, Gunoz H, Darendeliler F, Bas F (2006). Growth references for Turkish children aged 6 to 18 years. Acta Paediatr..

[10] Human Development Report 2011 Team (2011). Human development report 2011.

[11] UNICEF Zambia (2016). Annual report 2015.

[12] Central Statistical Office (2015). Zambia Demographic and Health Survey 2013-14.

[13] Weiner JS, Lourie JA (1981). Practical human biology.

[14] Cole TJ, Green PJ (1992). Smoothing references centile curves: the LMS method and penalized likelihood. Stat Med..

[15] Royston P, Wright EM (2000). Goodness-of-fit statistics for age-specific reference intervals. Stat Med..

[16] Pan H, Cole TJ (2004). A comparison of goodness of fit tests for age-related reference ranges. Stat Med..

[17] van Buuren S, Fredriks M (2001). Worm plot: a simple diagnostic device for modeling growth reference curves. Stat Med..

[18] Kuczmarski RJ (2002). 2000 CDC Growth Charts for the United States: methods and development. Vital Health Stat 11.

[19] WHO Working Group (1986). Use and interpretation of anthropometric indicators of nutritional status. Bull World Health Organ..

[20] Yamauchi T (2009). Growth and nutritional status of children and adults living in contrasting ecological zones in the Southern Province of Zambia. Vulnerability and resilience of social-ecological systems.

[21] Corlett JT (1986). Growth of urban schoolchildren in Botswana. Ann Hum Biol..

[22] Corlett JT, Woollard E (1988). Growth patterns of rural children in the Kgalagadi region of Botswana. Ann Hum Biol..

[23] Semproli S, Gualdi-Russo E (2007). Childhood malnutrition and growth in a rural area of Western Kenya. Am J Phys Anthropol..

[24] Monyeki KD (2000). Growth and nutritional status of rural South African children 3-10 years old: the Ellisras growth study. Am J Hum Biol..

[25] Sellen DW (1999). Growth patterns among seminomadic pastoralists (Datoga) of Tanzania. Am J Phys Anthropol..

[26] Olivieri F, Semproli S, Pettener D, Toselli S (2008). Growth and malnutrition of rural Zimbabwean children (6-17 years of age). Am J Phys Anthropol..

[27] Eliana MP, Kori BF, Alice SA (2004). Body mass index chart: useful yet underused. J Pediatr..

[28] Goon DT, Toriola AL, Shaw BS, Amusa LO, Monyeki MA, Akinyemi O (2011). Anthropometrically determined nutritional status of urban primary schoolchildren in Makurdi, Nigeria. BMC Public Health.

[29] Williams J, Best D (1982). Measuring sex stereotypes.

[30] Wanami H (2007). Boys are more stunted than girls in Sub-Saharan Africa: a meta-analysis of 16 demographic and health surveys. BMC Pediatr..

[31] Wells JCK (2000). Natural selection and sex differences in morbidity and mortality in early life. J Theor Biol..

[32] Central Statistical Office (1993). Zambia Demographic and Health Survey 1992.

[33] Cole TJ (2003). The secular trend in human physical growth: a biological view. Econ Hum Biol..

[34] Travaglino P, Meazza C, Pagani S, Biddeci G, Bozzola M (2011). Secular trends in growth of African pygmies and Bantu. Hormones..

[35] Gillett-Netting R, Meloy M, Campbell BC (2004). Catch-up reproductive maturation in rural Tonga girls, Zambia?. Am J Hum Biol..

[36] Campbell BC, Gillett-Netting R, Meloy M (2004). Timing of reproductive maturation in rural versus urban Tonga boy, Zambia. Ann Hum Biol.

[37] Kurz KM (1994). Adolescent growth. SCN News..

[38] Silventoinen K (2003). Determinants of variation in adult body height. J Biosoc Sci..

[39] Strategy and Policy Unit (2008). Zambia Millennium Development Goals Progress Report 2008.

[40] Takaishi M (1994). Secular changes in growth of Japanese children. J Pediatric Endocrinol.

[41] Maria EPR, Eyra ECB, Paola SL, Margarita DOC, Robert MM (2009). Growth status of indigenous school children 6–14 years in the Tarahumara Sierra, Northern Mexico, in 1990 and 2007. Ann Hum Biol..

[42] Zhen-Wang BI, Cheng-Ye JI (2005). Secular growth changes in body height and weight in children and adolescents in Shandong, China between 1939 and 2000. Ann Hum Biol..

[43] Jakimaviciene EM, Tutkuviene J (2007). Trends in body mass index, prevalence of overweight and obesity in preschool Lithuanian children, 1986-2006. Coll Antropol..

[44] dos Santos FK, Maia JAR, Gomes TNQF, Daca T, Madeira A, Katzmarzyk PT, et al. Secular trends in growth and nutritional status of Mozambican school-aged children and adolescents. PLoS One. 2014;9(12):e114068. 10.1371/journal.pone.0114068 PMC4256401 25473837

[45] Yamauchi T, Kon S. Variation in the nutritional status of adults living in contrasting ecological zones in the Southern Province of Zambia. In: Vulnerability and resilience of social-ecological systems: FY2009 FR3 Project Report; 2010: 45–5.

