# Supplementary material for: Evaluation of the nutritional status of rural children living in Zambia
Source: J Physiol Anthropol. 2020 Nov 16;39:34. doi: 10.1186/s40101-020-00244-8 (PMC7667821; doi:10.1186/s40101-020-00244-8)
Supplement: Supplementary file 1 — Additional file 1. [file 40101_2020_244_MOESM1_ESM.docx]

Answer Sheet

| From Reviewer 5 | |  |  |
| --- | --- | --- | --- |
| No | Comment from email | Comment | Answer |
| 1 |  | The article is written on a relevant topic, the research methodology and data are sufficient, but some adjustments should be made, which are indicated in the comments in the PDF file. | Thank you very much for your input. |
| No | Comment from PDF | Comment | Answer |
| 2 | Page 1, Line 3-4 | The title of this article does not exactly match the results presented: it is not the growth curves that are examined here, but the percentiles of some indicators are discussed and compared with other studies. In addition, the nutritional status is not considered here, but growth status. | Thank you very much for your suggestion.  Your point is right. We have revised as below.  Before: Development of a child growth chart and evaluation of the nutritional status of rural children living in Zambia  After: Evaluation of the growth status of rural children living in Zambia |
| 3 | Page 2, Line 46-47 | This is true for developed countries, however, children in developing countries with poor economic and health care system, usually are growing not according the optimal health status. The growth of these children is not optimal, so it is not recommended to develop a growth standard based on such growth indicators. On the other hand, the growth rates of many children in Northern Europe are well above those recommended by the WHO [here it is suggested to cite: Hermanussen, M., Aßmann, C., Tutkuvienė, J. (2010). Statistical agreement and cost-benefit: comparison of methods for constructing growth reference charts. Annals of human biology. ISSN 0301-4460. 37(1). 57-69.] | Thank you very much for your suggestion.  We understand your point and your point is right. We understand your point and it is right. However, in this study, our object was to confirm the position of our subject visually by comparing international standard and other sub-Saharan countries. We consider that to develop growth standard for our subject was the best way to compare with these references. |
| 4 | Page 2, Line 53 | The authors did not examine "nutritional status", only physical status - please avoid this term throughout the entire text of this article. | Thank you very much for your suggestion.  We have revised as below.  Before: at the following three points to evaluate the nutritional and growth status of …  After: at the following three points to evaluate the growth status of… |
| 5 | Page 2, Line 55-56 | However, Growth Charts are not provided at this study? | We have provided three smoothed growth curves of 5^th^, 50^th^ and 95^th^ percentiles for height and weight in Figure 1 and 2. Please kindly confirm “Figure” file. |
| 6 | Page 3, Line 55 | The contingent studied should be broken down by sex and age. | We have provided sex and age specific data on Table 2 and 3.  Please kindly let me know if my understanding is wrong. |
| 7 | Page 4, Line 6-7 | It is necessary to mention that other age groups were also determined according to this approach. | Thank you very much for your suggestion.  We have added below sentence.  “Other age group were also determined according to this approach.” |
| 8 | Page 4, Line 47-50 | Still, it remains unclear how the exact age of the children was determined? | Thank you very much for your suggestion.  We have added below sentence.  “Through this information, we could confirm the date of birth of the subjects and clarify the exact age of them.” |
| 9 | Page 5, Line 6-7 | However, the latter data are not provided in this article - could the authors provide them? In addition, it would be better use WHO Growth charts for comparisons. | As for smoothed growth curves for height and weight, we have provided in “Figure” file. Please kindly confirm.  As for comparison reference, thank you very much for your suggestion. Same question was already asked by reviewer 2 and we have made reply. We chose to uses the CDC US standard not the WHO standard because CDC reference has a strong advantage that can be used continuously from ages 2-19. In contrast the WHO growth charts only provide information on children up to 5 years of age. Of course, we understood that for children 2-5 years, WHO also has a growth chart and the methods used to create are similar between CDC growth chart and WHO growth chart. However, our subjects are 2-19 years like CDC, therefore, we have used CDC reference not WHO reference. |
| 10 | Page 5, Line 25 | These Figures are not provided at this version of manuscript? | We have provided “Figure” file and it had not revised since the first submission. As for detailed information, please kindly communicate handling editor. |
| 11 | Page 5, Line 28-29 | WHO growth curves are commonly used to compare data from different countries. | Please kindly confirm the answer No. 9. |
| 12 | Page 5, Line 33 | growth status or physical status | Thank you very much for your suggestion.  We have revised as below.  Before: nutritional status  After: growth status |
| 13 | Page 5, Line 56-58 | This statement cannot be clarified as the latter data are not provided - can the authors provide growth curves for these indicators? | We have provided growth curves for these indicators, please kindly confirm “Figure” file. |
| 14 | Page 6, Line 3-4 | However, the above data are not provided in Table 1 (?) | Thank you for pointing it out.  We have revised as below.  Before: (Tables 1 and 2)  After: (Table 2) |
| 15 | Page 6, Line 22 | These Figures are not provided at this version of this manuscript? | We have provided Figure 3 and 4, please kindly confirm “Figure” file. |
| 16 | Page 6, Line 50 | This statement is incorrect because nutrition is not addressed here | Thank you very much for your suggestion.  We have revised as below.  Before: In other words, although many subjects were small (stunting) and low weight (underweight), it was found that there were few poor nutritional statuses as judged by BMI.  After: In other words, although many subjects were small (stunting) and low weight (underweight), it was found that many subjects were normal as judged by BMI. |
| 17 | Page 7, Line 22 | This statement applies to countries with a high economic status and an advanced child health system. | Thank you very much for your suggestion.  We have revised as below.  Before: Local references best reflect the ethnic characteristics of a particular population  After: Local references best reflect the current ethnic characteristics of a particular population. |
| 18 | Page 7, Line 22-24 | However, local references were not provided in this paper. | Thank you for pointing it out.  We have provided local references in Figure 1 and 2.  In addition, we have revised this sentence as below.  Before: By developing and adapting local references, the growth and nutritional status of children…  After: By developing and adapting local references, the growth status of children…  Please kindly let me know if my understanding is wrong. |
| 19 | Page 7, Line 39 | Growth status | Thank you very much for your suggestion.  We have revised as below.  Before: Nutritional status from the growth chart and z-score  After: Growth status from the growth chart and z-score |
| 20 | Page 7, Line 42 | ??? | We have provided Figure 1 and 2, please kindly confirm “Figure” file. |
| 21 | Page 7, Line 47 | ??? | We have provided Figure 3 and 4, please kindly confirm “Figure” file. |
| 22 | Page 7, Line 53 | It is better to use WHO growth curves for comparisons. | Please kindly confirm the answer No. 9. |
| 23 | Page 7, Line 55-56 | Nutritional status was not investigated here - growth or physical status was investigated. | Thank you very much for your suggestion.  We have revised as below.  Before: nutritional status was not poor  After: growth status was not poor |
| 24 | Page 8, Line 6 | Growth status. | Thank you very much for your suggestion.  We have revised as below.  Before: Sex differences in nutritional status  After: Sex differences in growth status |
| 25 | Page 8, Line 9 | ??? | We have provided Figure 3 and 4, please kindly confirm “Figure” file. |
| 26 | Page 8, Line 17 | ??? | We have provided Figure 3 and 4, please kindly confirm “Figure” file. |
| 27 | Page 8, Line 25 | growth status | Thank you very much for your suggestion.  We have revised as below.  Before: nutritional status  After: growth status |
| 28 | Page 8, Line 52-53 | This phenomenon is evident almost in all ethnic populations, also in developed countries. | Thank you very much for your suggestion. Your point is right, however, since we would like to specialize in sub-Saharan Africa in this research, we cited an article which specialized in “stunted” situation in sub-Saharan Africa. |
| 29 | Page 10, Line 51-54 | Here it would be good to cite the following articles as well:  Tutkuviene, J. (2005).  Sex and gender differences in secular trend of body size and frame indices of Lithuanians. Anthropologischer Anzeiger. ISSN 0003-5548.63. 29-44.  Jakimaviciene, E.M., Tutkuviene, J. (2007). Trends in Body Mass Index, Prevalence of Overweight and Obesity in Preschool Lithuanian Children, 1986-2006. Collegium Antropologicum. ISSN 0350-6134. 31(1). 79-88.  Tutkuviene, J. (2007). Body Mass Index, Prevalence of Overweight and Obesity in Lithuanian Children and Adolescents, 1985-2002. Collegium Antropologicum. ISSN 0350-6134. 31(1). 109-121. | Thank you very much for your suggestion.  We have cited your recommended article also. |
| 30 | Page 11, Line 58 | I did not find these charts next to this manuscript? | Please kindly confirm “Figure” file. |
| 31 | Page 12, Line 4 | ??? | Thank you for pointing it out.  We have revised as below.  Before: The subjects were small and low weight compared to the US population, but the nutritional status was not poor but BMI.  After: The subjects were small and low weight compared to the US population, but the growth status was not poor as judged by BMI. |
| From Reviewer 1 | |  |  |
| No | Comment from PDF | Comment | Answer |
|  |  | I reviewed this paper earlier and explicitly mentioned the current debate on "stunting" and "malnutrition". The authors continue writing sentences like "Additionally, the nutritional status of the subjects was evaluated by determining the z-score." This is nonsense. The nutritional status of a child is NOT determined by the z-score of height. Stunting is not a synonym of malnutrition. I suggested to discuss the recent paper of Scheffler C et al. Stunting is not a synonym of malnutrition. Eur J Clin Nutr. 2019 May 29. doi: 10.1038/s41430-019-0439-4. This was not done. |  |
|  |  | Partially omitting the term "nutrition" from various sentences is NOT an answer to my comments. |  |
|  |  | I recommended showing the original raw data as smoothing eliminates much of the information, e.g. the adolescent growth spurt. Answering "our position is just to understand rough trends" is understandable but understanding rough trends cannot be the aim of a modern scientific paper. |  |
|  |  | When authors do not respond to reviewers' comments, I cannot see why a reviewer should review this manuscript again. |  |
